# Supplementary material for: Global Regulator PhoP is Necessary for Motility, Biofilm Formation, Exoenzyme Production, and Virulence of Xanthomonas citri Subsp. citri on Citrus Plants
Source: Genes (Basel). 2019 May 6;10(5):340. doi: 10.3390/genes10050340 (PMC6562643; doi:10.3390/genes10050340)
Supplement: Supplementary file 1 [file genes-10-00340-s001.zip › Fig and Supplementary/Table S1.docx]

**Table S1.** **Primers used in this study**

| primer | sequence（5’ –3’） | Size  (bp) | **applications** |
| --- | --- | --- | --- |
| *phoP*-U-F/  *phoP*-U-R | CGGGATCCTCCGATGCTCGACCTTCAAT/ CGGAATGGCGAAGCGATAACCGACCAAAAGGATACGCATG | 527 | fragement flanking the left of *phoP* gene |
| *phoP*-D-F/  *phoP*-D-R | CATGCGTATCCTTTTGGTCGGTTATCGCTTCGCCATTCCG/ CGGACTAGTCGCACCACTTCCACATACAG | 357 | fragement flanking the right of *phoP* gene |
| *phoP*-CX-F/  *phoP*-CX-R | GAGTATTTCCGGTCGCTTCT/ CCGTCGATCTGGGTCATTTC | 1790 | For *phoP* deletion sequencing |
| *phoP*-F/ *phoP*-R | CCAAGCTTGGGAGAGTGCATGCGTATCC/ CGGGATCCACTTGGTCTTCCTGCTCGTT | 630 | complementation fragment of *phoP* |
| 16S-qF/16S-qR | CGCTTTCGTGCCTCAGTGTCAGTGTTGG/ GGCGTAAAGCGTGCGTAGGTGGTGGTT |  | For qRT-PCR |
| *hrpG-*qF/ *hrpG-*qR | GCCTTTCAATTCGCACGAGTTACACG/ CACACGCCGGGGCTGGAAAAGA |  | For qRT-PCR |
| *hrpX*-qF/ *hrpX*-qR | AGCGATCTCTGCGTTGTCCTAC/ ATACGCATCTTCGGCCTCTTCCTGA |  | For qRT-PCR |
| *hrcN-*qF/ *hrcN-*qR | CGAGGTGCGGGAGTTCATC / ATGGCGGTGCCGACGTAAG |  | For qRT-PCR |
| *hrcQ-*qF/ *hrcQ-*qR | ATCGGCATCGGCAGGTTAGTG/ CATGCGTGCTCTCCGACAT |  | For qRT-PCR |
| *avrBs2-*qF/ *avrBs2-*qR | CGCGCCAATCACGACAAGGACTACTAC/ CGGGCCAGCGTGCGGTTTTC |  | For qRT-PCR |
| *avrXacE1-*qF/ *avrXacE1-*qR | TCGCGCTGGGCCGGAACATACC/ GCGTCCGCGGCGATAACTCTTG |  | For qRT-PCR |
| *pthA-*qF/ *pthA-*qR | TGGCGTCGGCAAACAGTGGTC/ TGCTCCGGGGTCAGGTTCAGG |  | For qRT-PCR |
| *egl 0028-*qF/  *egl 0028-*qR | GTATTCCACCACGAACGA/ CGGCATCCAGATACTGAT |  | For qRT-PCR |
| *cheA-*qF/ *cheA-*qR | CGAAATCGCCCGCAACAA/ CGGTGACTTCCGCCTTGG |  | For qRT-PCR |
| *cheY-*qF/ *cheY-*qR | CGTCAAGAATCTGTTGGGCGA/ CCAGTCGGTGACCACGAAATC |  | For qRT-PCR |
| *fliC-*qF/ *fliC-*qR | GCCAGACCATCGGCATCA/ ACGGCGGCAGCGAAGTTG |  | For qRT-PCR |
| *flhF-*qF/ *flhF-*qR | CGCTGCCGATAAGCCCTGTA/ GGTTGGCGGAGATGTTGGTT |  | For qRT-PCR |
| *pqqG-*qF/ *pqqG-*qR | GGCGTGGCAGCAAACAGA/ GTTGGCGGAAATCGGGAG |  | For qRT-PCR |
| *rpoN-*qF/ *rpoN-*qR | CGTGGGGTCTGGATGTAC/ GCTGACGCTGAAGGAAAT |  | For qRT-PCR |
| *XacPNP*-qF/ *XacPNP*-qR | TTAGTGTCGCAGCAATCG/ ACAAGAAGCACCGTTATCC |  | For qRT-PCR |
| *virB1*-qF/ *virB1*-qR | GTAATCCCTACGCCATTG/ TTGTAACCCTTCTGCTCC |  | For qRT-PCR |
