# Supplementary material for: Global Regulator PhoP is Necessary for Motility, Biofilm Formation, Exoenzyme Production, and Virulence of Xanthomonas citri Subsp. citri on Citrus Plants
Source: Genes (Basel). 2019 May 6;10(5):340. doi: 10.3390/genes10050340 (PMC6562643; doi:10.3390/genes10050340)
Supplement: Supplementary file 1 [file genes-10-00340-s001.zip › Fig and Supplementary/Table S2 Statistics of reads map to reference genome.docx]

**Table S2 Statistics of reads map to reference genome**

| Sample name | △*phoP* | XHG3 |
| --- | --- | --- |
| Total reads | 26469538 | 19945236 |
| Total mapped Reads | 24370669 (92.07%) | 19124608 (95.89%) |
| Multiple mapped | 152024 (0.57%) | 86153 (0.43%) |
| Uniquely mapped | 24218645 (91.5%) | 19038455 (95.45%) |
| Read-1 | 12028572 (45.44%) | 9471075 (47.49%) |
| Read-2 | 12190073 (46.05%) | 9567380 (47.97%) |
| Reads map to '+' | 12127260 (45.82%) | 9527821 (47.77%) |
| Reads map to '-' | 12091385 (45.68%) | 9510634 (47.68%) |
| Reads mapped in proper pairs | 191477 (0.72%) | 147063 (0.74%) |

(1) Total reads：Number of sequencing sequences filtered by sequencing data (Clean data)

(2) Total mapped：number of sequences that can be mapped on a genome;In general, the percentage is more than 70%.

(3) Multiple mapped：Statistics of the sequencing sequences with multiple alignment locations on the reference sequences; The percentage is typically less than 10%.

(4) Uniquely mapped：Number of sequencing sequences with a unique alignment on the reference sequence.

(5) Reads map to '+'，Reads map to '-'：Statistics of sequencing sequences were compared to positive and negative chains on the genome
