# Supplementary material for: Global Regulator PhoP is Necessary for Motility, Biofilm Formation, Exoenzyme Production, and Virulence of Xanthomonas citri Subsp. citri on Citrus Plants
Source: Genes (Basel). 2019 May 6;10(5):340. doi: 10.3390/genes10050340 (PMC6562643; doi:10.3390/genes10050340)
Supplement: Supplementary file 1 [file genes-10-00340-s001.zip › Fig and Supplementary/Table S3.docx]

**Table S3. △*phoP* vs XHG3 difference genes by RNA-seq (∣log_2_.Fold_change∣≧1.2)**

| Gene_id | log_2_.Fold_change | p.value | Gene Name |
| --- | --- | --- | --- |
| XAC2788 | 6.5526 | 2.05E-81 | *GN=yuzA* |
| XAC2155 | 6.3435 | 0 | *GN=yciE* |
| Novel00038 | 6.3148 | 2.28E-06 | *-//-* |
| sRNA00033 | 4.9662 | 1.59E-70 | *-//-* |
| XAC0525 | 4.926 | 7.65E-26 | *-//-* |
| XAC2368 | 4.8373 | 1.61E-29 | *GN=Mb2322c* |
| XAC1193 | 4.5993 | 3.36E-87 | *-//-* |
| XAC0098 | 4.5746 | 5.39E-43 | *-//-* |
| XAC3740 | 4.5127 | 2.58E-19 | *GN=galE* |
| XAC3545 | 4.4906 | 0 | *GN=XCC0851* |
| sRNA00016 | 4.1887 | 4.25E-31 | *-//-* |
| XAC0231 | 4.0612 | 8.67E-17 | *-//-* |
| XAC1192 | 3.9827 | 4.54E-30 | *GN=tcmJ* |
| XAC2024 | 3.9409 | 4.59E-47 | *-//-* |
| XAC4275 | 3.914 | 1.90E-23 | *GN=NGR_a00820* |
| XAC2027 | 3.8976 | 2.03E-31 | *-//-* |
| XAC3964 | 3.8689 | 2.22E-255 | *GN=AF_1420* |
| sRNA00006 | 3.8649 | 5.59E-25 | *-//-* |
| XAC1189 | 3.8318 | 2.49E-63 | *GN=yagT* |
| XAC0834 | 3.8249 | 0 | *GN=mprA* |
| XAC2028 | 3.7302 | 1.26E-59 | *GN=fdh* |
| XAC3204 | 3.7195 | 1.25E-62 | *-//-* |
| XAC3725 | 3.7009 | 0 | *GN=yciF* |
| XAC0097 | 3.6882 | 2.20E-73 | *-//-* |
| sRNA00060 | 3.5895 | 3.32E-07 | *-//-* |
| XAC2139 | 3.528 | 1.85E-27 | *-//-* |
| XAC4274 | 3.5249 | 1.25E-131 | *GN=oar* |
| XAC2026 | 3.5235 | 8.47E-77 | *-//-* |
| XAC1548 | 3.4725 | 4.94E-41 | *GN=yhcF* |
| XAC0747 | 3.4685 | 8.98E-87 | *-//-* |
| XAC0358 | 3.4667 | 1.56E-107 | *GN=glpK* |
| XAC1188 | 3.45 | 3.64E-49 | *GN=yagS* |
| XAC1818 | 3.4375 | 0.00037891 | *GN=fhaB* |
| XAC2844 | 3.435 | 3.13E-163 | *GN=mexA* |
| XAC1819 | 3.3496 | 2.26E-14 | *GN=crtK* |
| XAC1178 | 3.3218 | 1.34E-125 | *GN=yhxD* |
| XAC3203 | 3.2543 | 1.73E-32 | *GN=GST* |
| sRNA00005 | 3.2359 | 6.00E-08 | *-//-* |
| XAC4318 | 3.2144 | 3.10E-41 | *-//-* |
| XAC1632 | 3.1396 | 9.31E-35 | *-//-* |
| XAC0190 | 3.1323 | 1.32E-70 | *-//-* |
| XAC3746 | 3.0984 | 1.14E-111 | *-//-* |
| XAC2920 | 3.0779 | 2.15E-49 | *GN=yecE* |
| XAC3732 | 3.076 | 8.55E-18 | *-//-* |
| XAC0110 | 3.0338 | 7.30E-43 | *GN=ousA* |
| XAC0585 | 3.0142 | 2.21E-38 | *GN=yqjG* |
| XAC0096 | 2.994 | 6.06E-134 | *-//-* |
| XAC2367 | 2.9928 | 2.30E-55 | *-//-* |
| XAC2188 | 2.9856 | 3.97E-21 | *-//-* |
| sRNA00058 | 2.98 | 0.00029355 | *-//-* |
| XAC1443 | 2.9794 | 4.73E-96 | *GN=ydgJ* |
| XAC2415 | 2.9737 | 7.04E-36 | *-//-* |
| XAC0755 | 2.9421 | 4.35E-08 | *-//-* |
| XAC3738 | 2.938 | 1.21E-56 | *GN=yghA* |
| XAC1180 | 2.9371 | 3.29E-20 | *GN=hetN* |
| XAC2613 | 2.9044 | 4.72E-283 | *-//-* |
| XAC2617 | 2.9025 | 8.15E-188 | *GN=virB1* |
| XAC2895 | 2.8922 | 2.04E-127 | *GN=yagT* |
| XAC2138 | 2.8854 | 8.94E-56 |  |
| XAC3734 | 2.8816 | 2.41E-27 | *-//-* |
| XAC2616 | 2.8675 | 0 | *-//-* |
| XAC0587 | 2.8668 | 1.58E-231 | *-//-* |
| XAC2742 | 2.8312 | 1.25E-243 | *GN=btuB* |
| XAC2025 | 2.823 | 1.65E-55 | *-//-* |
| XAC3096 | 2.8193 | 3.52E-21 | *-//-* |
| XAC3037 | 2.8109 | 6.19E-227 | *GN=bphD* |
| XAC2907 | 2.7978 | 1.95E-54 | *GN=K02A2.1* |
| XAC1174 | 2.7969 | 5.25E-27 | *GN=K02B2.1* |
| XAC0250 | 2.7934 | 5.09E-74 | *GN=Mb0930* |
| XAC3546 | 2.7898 | 0 | *GN=yadA* |
| XAC0099 | 2.7884 | 3.11E-12 | *-//-* |
| XAC2878 | 2.7726 | 7.78E-46 | *-//-* |
| XAC0131 | 2.7719 | 2.51E-17 | *-//-* |
| XAC2663 | 2.7583 | 8.03E-08 | *-//-* |
| XAC4351 | 2.7418 | 1.41E-08 | *-//-* |
| XAC0740 | 2.7405 | 1.02E-49 | *-//-* |
| XAC1444 | 2.7316 | 0 | *GN=oprM* |
| XAC1521 | 2.7216 | 2.46E-173 | *GN=grpE* |
| Novel00018 | 2.7059 | 3.70E-27 | *-//-* |
| XAC2923 | 2.692 | 1.25E-76 | *GN=pilT* |
| XAC0544 | 2.6861 | 3.63E-40 | *GN=btuB* |
| XAC0035 | 2.6825 | 6.15E-82 | *GN=TOP1E* |
| XAC2992 | 2.6663 | 0 | *GN=prpL* |
| XAC3101 | 2.6514 | 3.89E-22 | *GN=pilH* |
| XAC0921 | 2.6458 | 7.75E-07 | *-//-* |
| XAC3970 | 2.6417 | 1.18E-186 | *-//-* |
| XAC0637 | 2.6366 | 1.70E-121 | *GN=hslV* |
| XAC3684 | 2.628 | 6.95E-24 | *-//-* |
| XAC0582 | 2.6259 | 2.71E-35 | *-//-* |
| XAC0455 | 2.6187 | 1.76E-39 | *-//-* |
| XAC2064 | 2.6179 | 8.69E-33 | *GN=nolF* |
| Novel00040 | 2.5929 | 6.38E-33 | *-//-* |
| XAC2615 | 2.5723 | 0 | *GN=ptlB* |
| XAC3685 | 2.5657 | 4.03E-61 | *-//-* |
| XAC3747 | 2.5453 | 1.09E-73 | *GN=ybdR* |
| XAC4259 | 2.5401 | 4.32E-89 | *GN=blc* |
| XAC3969 | 2.5394 | 3.24E-40 | *-//-* |
| XAC3100 | 2.5384 | 5.89E-18 | *GN=pilI* |
| XAC3728 | 2.513 | 3.88E-24 | *-//-* |
| XAC1547 | 2.503 | 3.65E-27 | *GN=yhcG* |
| XAC2152 | 2.497 | 1.39E-134 | *GN=NGR_a02630* |
| XAC3726 | 2.4957 | 1.76E-115 | *GN=CA_C2800* |
| XAC3382 | 2.4955 | 2.35E-24 | *-//-* |
| Novel00001 | 2.4923 | 5.14E-49 | *-//-* |
| XAC3632 | 2.4851 | 4.78E-39 | *GN=gloA* |
| XAC0835 | 2.4848 | 2.99E-147 | *GN=qseC* |
| XAC2884 | 2.4792 | 8.22E-50 | *GN=phlB* |
| XAC0584 | 2.4734 | 1.88E-05 | *-//-* |
| XAC1187 | 2.4724 | 1.99E-87 | *GN=yagR* |
| XAC3097 | 2.467 | 9.06E-64 | *GN=cheB1* |
| XAC3560 | 2.4656 | 5.46E-134 | *GN=btuB* |
| XAC1186 | 2.4655 | 3.67E-66 | *GN=uvrA* |
| XAC0606 | 2.4515 | 6.43E-32 | *GN=Neil3* |
| XAC0158 | 2.4501 | 7.24E-148 | *GN=pld1* |
| XAC3727 | 2.4467 | 4.52E-28 | *-//-* |
| XAC3777 | 2.4417 | 6.71E-17 | *-//-* |
| XAC2932 | 2.435 | 9.54E-132 | *GN=yraA* |
| XAC0107 | 2.4319 | 4.55E-33 | *-//-* |
| XAC3844 | 2.4278 | 3.99E-39 | *-//-* |
| XAC3741 | 2.4214 | 9.82E-12 | *GN=tuaH* |
| XAC0360 | 2.4142 | 1.34E-114 | *GN=glpD* |
| XAC4114 | 2.4138 | 7.86E-08 | *GN=shlB* |
| XAC2891 | 2.4094 | 5.88E-05 | *GN=yqeB* |
| XAC2793 | 2.4054 | 9.57E-84 | *-//-* |
| XAC2924 | 2.3947 | 9.51E-117 | *GN=pilT* |
| XAC3351 | 2.3795 | 9.40E-75 | *-//-* |
| XAC2843 | 2.3787 | 3.38E-76 | *GN=mexB* |
| XAC2508 | 2.3558 | 4.89E-07 | *-//-* |
| XAC2031 | 2.355 | 1.75E-09 | *-//-* |
| XAC1642 | 2.3547 | 4.46E-25 | *-//-* |
| XAC1665 | 2.349 | 1.54E-22 | *-//-* |
| XAC1355 | 2.3469 | 2.73E-37 | *GN=ybiI* |
| XAC2614 | 2.3377 | 1.57E-258 | *GN=virB4* |
| XAC0922 | 2.3349 | 2.23E-33 | *-//-* |
| XAC1177 | 2.3297 | 1.63E-23 | *GN=MSMEG_4535* |
| XAC3733 | 2.3276 | 1.87E-216 | *GN=ntrC* |
| XAC0239 | 2.3236 | 0 | *GN=XOO4217* |
| XAC3956 | 2.3231 | 9.62E-17 | *GN=blc* |
| XAC2127 | 2.3161 | 0.00078415 | *-//-* |
| XAC3050 | 2.3155 | 0 | *GN=btuB* |
| XAC1869 | 2.3048 | 5.29E-50 | *-//-* |
| XAC3802 | 2.3048 | 1.23E-128 | *GN=xkdP* |
| XAC1870 | 2.3033 | 1.52E-09 | *-//-* |
| XAC0794 | 2.2989 | 1.44E-46 | *GN=TP53I3* |
| XAC3380 | 2.2951 | 0 | *-//-* |
| XAC0638 | 2.2905 | 7.90E-213 | *GN=hslU* |
| XAC1868 | 2.2832 | 9.79E-11 | *GN=wapA* |
| XAC2733 | 2.2801 | 6.67E-12 | *-//-* |
| XAC3865 | 2.2787 | 1.24E-15 | *-//-* |
| XAC2655 | 2.2715 | 3.71E-06 | *GN=J* |
| XAC0251 | 2.2571 | 4.63E-46 | *GN=rutR* |
| XAC3502 | 2.2571 | 6.63E-06 | *-//-* |
| XAC3381 | 2.2529 | 3.38E-147 | *GN=pilQ* |
| sRNA00044 | 2.2502 | 4.73E-08 | *-//-* |
| XAC1633 | 2.2488 | 4.35E-72 | *GN=sldA* |
| XAC0467 | 2.2437 | 2.64E-52 | *-//-* |
| XAC3127 | 2.2431 | 0.00012038 | *-//-* |
| XAC2154 | 2.2416 | 6.21E-30 | *-//-* |
| XAC2612 | 2.2407 | 6.92E-45 | *-//-* |
| XAC0830 | 2.2359 | 0.00028075 | *GN=tauD* |
| XAC3745 | 2.2344 | 1.18E-17 | *-//-* |
| XAC2136 | 2.2286 | 0.00013759 | *GN=DHDH* |
| XAC3981 | 2.2284 | 9.64E-198 | *-//-* |
| XAC4205 | 2.2238 | 0.0032339 | *-//-* |
| XAC0547 | 2.2237 | 5.19E-26 | *-//-* |
| XAC0506 | 2.2172 | 5.14E-54 |  |
| XAC0466 | 2.2167 | 8.26E-106 | *GN=HI_1415* |
| Novel00006 | 2.2135 | 1.65E-05 | *-//-* |
| XAC3845 | 2.2116 | 2.94E-36 | *GN=ygaU* |
| XAC2065 | 2.1923 | 9.12E-29 | *GN=nolG* |
| XAC2619 | 2.1917 | 6.58E-139 | *GN=virB10* |
| XAC3098 | 2.1871 | 9.39E-232 | *GN=frzE* |
| XAC2618 | 2.1805 | 5.56E-99 | *GN=virB11* |
| XAC1398 | 2.1787 | 1.21E-09 | *GN=ywzG* |
| XAC3739 | 2.1759 | 3.17E-52 | *-//-* |
| XAC3242 | 2.1712 | 7.43E-132 | *GN=pilC* |
| XAC2894 | 2.1674 | 1.68E-54 | *GN=yagS* |
| XAC2930 | 2.1643 | 5.37E-11 | *-//-* |
| XAC1387 | 2.164 | 3.47E-12 | *-//-* |
| XAC1062 | 2.1633 | 0 | *-//-* |
| XAC3972 | 2.1592 | 7.60E-92 | *-//-* |
| XAC0839 | 2.1454 | 4.98E-35 | *GN=ygcG* |
| XAC3259 | 2.1453 | 3.85E-08 | *-//-* |
| XAC0682 | 2.1426 | 0 | *GN=osmY* |
| XAC2301 | 2.1287 | 8.90E-44 | *-//-* |
| XAC4139 | 2.1283 | 5.69E-06 | *-//-* |
| XAC1707 | 2.1264 | 7.48E-83 | *GN=bli-3* |
| XAC0323 | 2.1168 | 2.23E-26 | *-//-* |
| XAC0920 | 2.1133 | 1.46E-16 | *-//-* |
| XAC2369 | 2.1103 | 6.42E-62 | *-//-* |
| XAC1445 | 2.1096 | 1.00E-134 | *GN=emrA* |
| XAC4310 | 2.105 | 3.20E-21 | *GN=cls2* |
| XAC3634 | 2.0981 | 9.38E-43 | *GN=cwlL* |
| XAC0941 | 2.0968 | 1.33E-56 |  |
| XAC2819 | 2.0839 | 4.41E-15 | *-//-* |
| XAC1554 | 2.083 | 1.50E-51 | *-//-* |
| XAC1778 | 2.082 | 2.26E-69 | *-//-* |
| XAC1200 | 2.0793 | 5.37E-34 | *GN=dpf-6* |
| XAC0016 | 2.0761 | 9.66E-10 | *-//-* |
| XAC0359 | 2.0702 | 1.38E-32 | *GN=glpF* |
| XAC0356 | 2.0671 | 8.79E-08 | *GN=pobA* |
| XAC2622 | 2.0619 | 5.99E-72 | *-//-* |
| XAC2030 | 2.0556 | 4.02E-31 | *GN=xthA* |
| Novel00041 | 2.0548 | 1.06E-21 | *-//-* |
| XAC0132 | 2.0507 | 2.17E-40 | *GN=COXBURSA331_A2134* |
| XAC4233 | 2.0478 | 4.71E-06 | *GN=ble* |
| XAC2469 | 2.0442 | 5.29E-68 | *GN=sad* |
| XAC2885 | 2.0402 | 1.60E-42 | *GN=phlA* |
| XAC0548 | 2.0373 | 1.13E-07 | *GN=gnl* |
| XAC4353 | 2.0285 | 2.27E-31 | *-//-* |
| XAC0621 | 2.0238 | 1.54E-22 | *GN=algR* |
| XAC2664 | 2.0073 | 7.91E-14 | *-//-* |
| XAC2057 | 2.0053 | 7.19E-41 | *-//-* |
| XAC3383 | 2.0008 | 6.23E-37 | *-//-* |
| XAC0109 | 1.9961 | 3.52E-09 | *GN=trxA* |
| XAC0518 | 1.9929 | 3.18E-06 | *-//-* |
| XAC0469 | 1.9908 | 0.0032564 | *-//-* |
| XAC3691 | 1.9883 | 3.48E-33 | *GN=msrB* |
| XAC3385 | 1.9878 | 2.07E-59 | *-//-* |
| XAC3866 | 1.9877 | 5.92E-163 | *-//-* |
| XAC2888 | 1.9851 | 5.04E-07 | *-//-* |
| XAC1487 | 1.979 | 1.27E-13 | *-//-* |
| XAC3776 | 1.9672 | 1.94E-13 | *-//-* |
| XAC2050 | 1.9657 | 1.43E-14 | *-//-* |
| XAC3971 | 1.9582 | 0 | *-//-* |
| XAC3692 | 1.9503 | 1.35E-17 | *-//-* |
| XAC1670 | 1.9497 | 5.20E-48 | *GN=PSPPH_2483* |
| XAC0840 | 1.949 | 2.25E-08 | *-//-* |
| XAC2557 | 1.9463 | 3.78E-52 | *GN=con-10* |
| XAC0515 | 1.9421 | 3.73E-12 | *GN=ynbC* |
| XAC2623 | 1.94 | 1.22E-115 | *GN=virD4* |
| XAC3195 | 1.9329 | 1.52E-224 | *GN=clpB* |
| XAC3926 | 1.9285 | 1.23E-09 | *-//-* |
| XAC2114 | 1.9183 | 4.35E-15 | *GN=zupT* |
| XAC0364 | 1.914 | 0.00012715 | *GN=catI* |
| XAC3099 | 1.9134 | 6.23E-76 | *GN=pilJ* |
| XAC2784 | 1.9103 | 2.63E-17 | *-//-* |
| XAC1349 | 1.9044 | 5.52E-58 |  |
| XAC3574 | 1.9027 | 1.56E-13 | *GN=Alkbh2* |
| XAC0541 | 1.8974 | 0 | *GN=groS* |
| XAC2873 | 1.8967 | 2.31E-10 | *-//-* |
| XAC3240 | 1.888 | 6.15E-22 | *GN=tfpQ* |
| XAC4008 | 1.8878 | 0 | *GN=ecnA* |
| XAC3748 | 1.8861 | 3.41E-19 | *-//-* |
| XAC4172 | 1.8859 | 0.00046278 | *GN=NGR_a03650* |
| XAC1175 | 1.8779 | 2.61E-09 | *GN=nnrD* |
| Novel00036 | 1.8774 | 2.13E-15 | *-//-* |
| XAC0287 | 1.8761 | 2.61E-21 | *GN=curA* |
| XAC0888 | 1.8745 | 4.20E-82 | *GN=gfo* |
| XAC3775 | 1.8733 | 0.00040311 | *-//-* |
| XAC2122 | 1.8727 | 4.18E-15 | *-//-* |
| XAC0468 | 1.8711 | 2.14E-22 | *-//-* |
| XAC2875 | 1.868 | 3.68E-28 | *GN=nfi* |
| XAC2621 | 1.8621 | 2.10E-93 | *GN=virB8* |
| XAC3843 | 1.8615 | 0.00035106 | *GN=cas2-3* |
| XAC1359 | 1.8526 | 1.05E-19 | *GN=slc30a9* |
| XAC0862 | 1.8492 | 4.37E-22 | *GN=apaG* |
| XAC0928 | 1.8468 | 5.88E-20 | *GN=XCC0851* |
| XAC3095 | 1.8425 | 9.71E-06 | *GN=rimJ* |
| XAC2732 | 1.8422 | 7.90E-10 | *-//-* |
| XAC4373 | 1.8367 | 1.53E-18 | *GN=rnpA* |
| XAC1629 | 1.8358 | 4.99E-27 | *-//-* |
| XAC3566 | 1.8229 | 2.76E-10 | *-//-* |
| XAC2137 | 1.8217 | 1.04E-11 | *GN=DHDH* |
| XAC0157 | 1.817 | 0.0010503 | *GN=MTH_1774* |
| XAC2620 | 1.8114 | 1.23E-90 | *GN=ptlF* |
| XAC4044 | 1.8114 | 1.35E-42 | *-//-* |
| XAC2880 | 1.8105 | 1.43E-43 | *GN=CC_0481* |
| XAC3133 | 1.8095 | 1.35E-47 | *GN=Mb2008* |
| XAC1307 | 1.7969 | 1.41E-08 | *-//-* |
| XAC3040 | 1.7858 | 5.75E-06 | *GN=metX* |
| XAC1321 | 1.7857 | 6.60E-224 | *GN=mucD* |
| XAC1454 | 1.7831 | 1.45E-21 | *-//-* |
| XAC2151 | 1.7723 | 0 |  |
| XAC1353 | 1.7685 | 2.76E-70 | *GN=XC_2938* |
| XAC2609 | 1.7682 | 2.55E-135 | *-//-* |
| XAC4041 | 1.7641 | 2.33E-22 | *-//-* |
| XAC3358 | 1.7632 | 1.14E-20 | *GN=modA* |
| XAC0516 | 1.7619 | 6.62E-05 | *GN=ynbD* |
| XAC2498 | 1.7545 | 1.45E-75 | *GN=acrA* |
| XAC0542 | 1.7489 | 0 | *GN=groL* |
| XAC1150 | 1.7445 | 4.19E-33 | *GN=PRXIIE-1* |
| XAC3513 | 1.7396 | 1.85E-39 | *-//-* |
| XAC1476 | 1.7381 | 4.71E-51 | *GN=yieF* |
| XAC1764 | 1.7373 | 1.82E-13 | *GN=araB* |
| XAC1388 | 1.7304 | 5.51E-28 | *-//-* |
| XAC0112 | 1.7259 | 6.22E-35 | *-//-* |
| sRNA00045 | 1.7233 | 7.08E-19 | *-//-* |
| XAC1346 | 1.7141 | 7.49E-11 | *-//-* |
| XAC2849 | 1.714 | 9.08E-11 | *GN=yhcX* |
| XAC4162 | 1.7101 | 0.00021686 | *GN=czcC* |
| XAC1183 | 1.7091 | 0.00039576 | *-//-* |
| XAC0605 | 1.7062 | 6.90E-06 | *GN=yecE* |
| XAC2745 | 1.7054 | 2.99E-125 | *GN=Ece1* |
| XAC1203 | 1.7024 | 6.04E-05 | *-//-* |
| XAC0422 | 1.7021 | 5.56E-62 | *GN=slr0889* |
| XAC2874 | 1.6998 | 4.81E-70 | *GN=gpmA* |
| XAC1389 | 1.6964 | 7.21E-15 | *GN=yxlF* |
| XAC3965 | 1.6941 | 3.28E-48 | *GN=rtcB* |
| XAC3199 | 1.692 | 1.58E-06 | *GN=gpr* |
| XAC4352 | 1.6918 | 6.47E-10 | *GN=gstB* |
| XAC2480 | 1.6906 | 6.96E-14 | *GN=puuA* |
| XAC3743 | 1.6903 | 3.97E-05 | *-//-* |
| XAC2610 | 1.6902 | 3.98E-128 | *-//-* |
| XAC0100 | 1.6873 | 1.47E-136 | *GN=XAC0100* |
| XAC0128 | 1.6833 | 3.97E-10 | *GN=ydbB* |
| XAC1438 | 1.6766 | 9.43E-92 | *GN=bfr* |
| XAC0586 | 1.6712 | 9.01E-14 | *-//-* |
| XAC0224 | 1.6688 | 1.65E-35 | *GN=poxB* |
| XAC3209 | 1.6617 | 1.16E-60 | *GN=otsB* |
| XAC2605 | 1.6607 | 6.60E-06 | *-//-* |
| XAC0942 | 1.6591 | 7.60E-15 | *GN=moaB* |
| XAC2187 | 1.6588 | 2.29E-08 | *-//-* |
| XAC1917 | 1.6581 | 9.62E-07 | *-//-* |
| XAC1381 | 1.6538 | 0.0035166 | *-//-* |
| sRNA00070 | 1.6536 | 1.42E-11 | *-//-* |
| XAC2528 | 1.6524 | 1.14E-262 | *GN=htpG* |
| XAC3211 | 1.6522 | 4.81E-107 | *GN=otsA* |
| XAC3434 | 1.6499 | 6.78E-12 | *GN=yneE* |
| XAC3711 | 1.6473 | 0.00023863 | *-//-* |
| XAC1242 | 1.6462 | 2.04E-17 | *-//-* |
| XAC3308 | 1.6453 | 1.15E-79 | *GN=mscL* |
| XAC2764 | 1.6451 | 1.03E-22 | *-//-* |
| XAC3384 | 1.6413 | 1.96E-27 | *-//-* |
| XAC0868 | 1.64 | 0 | *-//-* |
| sRNA00053 | 1.6254 | 0.00030002 | *-//-* |
| XAC2606 | 1.6202 | 0.00044797 | *-//-* |
| XAC1918 | 1.6201 | 1.15E-12 | *-//-* |
| XAC3607 | 1.6163 | 5.29E-47 | *GN=pilT* |
| XAC2667 | 1.6144 | 2.81E-14 | *-//-* |
| XAC0615 | 1.6125 | 8.94E-35 | *GN=Lema_P049800* |
| XAC0211 | 1.6116 | 4.86E-10 | *GN=fosB* |
| XAC4273 | 1.5952 | 1.76E-199 | *GN=oar* |
| XAC2719 | 1.5898 | 2.42E-26 | *GN=trpF* |
| XAC0108 | 1.587 | 3.23E-13 | *-//-* |
| XAC4182 | 1.5861 | 9.41E-56 | *-//-* |
| XAC0320 | 1.5846 | 7.12E-09 | *GN=cobB* |
| XAC2559 | 1.5844 | 1.36E-31 | *-//-* |
| XAC3665 | 1.5823 | 3.45E-05 | *-//-* |
| XAC2153 | 1.5812 | 1.34E-21 | *GN=REE1* |
| XAC1354 | 1.5766 | 7.55E-16 | *-//-* |
| XAC2797 | 1.5639 | 1.54E-36 | *GN=HI_1051* |
| XAC2872 | 1.5635 | 7.18E-21 | *GN=Ece1* |
| XAC3166 | 1.5633 | 7.19E-27 | *GN=btuB* |
| XAC1817 | 1.5622 | 2.50E-33 | *-//-* |
| XAC3422 | 1.5618 | 2.08E-18 |  |
| XAC1154 | 1.559 | 4.24E-17 | *GN=pilH* |
| Novel00009 | 1.5588 | 2.03E-17 | *-//-* |
| XAC2125 | 1.5508 | 0.00054114 | *-//-* |
| XAC3025 | 1.5501 | 1.05E-07 | *-//-* |
| XAC2156 | 1.5478 | 2.18E-89 | *-//-* |
| XAC1358 | 1.5476 | 5.70E-27 | *GN=slyD* |
| XAC0461 | 1.5407 | 0.0017709 | *GN=phaC* |
| XAC2818 | 1.5379 | 1.98E-06 | *-//-* |
| XAC2783 | 1.5367 | 2.50E-52 | *GN=ybbN* |
| XAC3044 | 1.5227 | 2.55E-40 | *GN=yhjG* |
| XAC3368 | 1.5224 | 1.26E-09 | *GN=lytH* |
| XAC0838 | 1.5213 | 1.96E-65 | *GN=lemA* |
| XAC2712 | 1.5191 | 2.50E-20 | *-//-* |
| XAC3102 | 1.5167 | 1.79E-54 | *GN=pilG* |
| XAC2666 | 1.5163 | 9.56E-05 | *-//-* |
| Novel00017 | 1.5105 | 5.45E-24 | *-//-* |
| XAC0549 | 1.5102 | 3.62E-09 | *-//-* |
| XAC4113 | 1.5076 | 1.67E-11 | *GN=ompA* |
| XAC2529 | 1.4999 | 1.08E-61 | *GN=rhsD* |
| XAC2665 | 1.4993 | 1.06E-43 | *-//-* |
| XAC0832 | 1.4972 | 1.14E-09 | *GN=ybbA* |
| XAC1433 | 1.4965 | 5.01E-31 | *GN=asnB* |
| XAC3922 | 1.496 | 3.34E-11 | *GN=dhbF* |
| XAC1106 | 1.4953 | 2.28E-05 | *-//-* |
| XAC1545 | 1.4891 | 3.44E-07 | *-//-* |
| XAC3031 | 1.4886 | 4.33E-11 | *GN=rpfC* |
| XAC1546 | 1.4873 | 3.46E-11 | *-//-* |
| XAC3491 | 1.4861 | 1.87E-12 | *GN=HSP31* |
| XAC3210 | 1.4794 | 5.87E-90 | *GN=Rv2402* |
| XAC1541 | 1.4749 | 1.01E-25 | *GN=yhdT* |
| XAC0081 | 1.4729 | 0.00014214 | *-//-* |
| XAC0036 | 1.472 | 1.62E-20 | *-//-* |
| XAC2711 | 1.4714 | 4.48E-16 | *GN=PA1727* |
| XAC0118 | 1.4705 | 9.00E-08 | *GN=yeaC* |
| XAC0288 | 1.4703 | 2.05E-40 | *GN=yrpG* |
| XAC1544 | 1.4683 | 9.48E-08 | *-//-* |
| XAC3633 | 1.4666 | 1.40E-50 | *-//-* |
| XAC3682 | 1.4666 | 3.65E-23 | *-//-* |
| XAC2439 | 1.4662 | 2.43E-20 | *GN=NGR_a03330* |
| XAC2463 | 1.4649 | 1.39E-11 | *-//-* |
| XAC0545 | 1.4601 | 1.35E-16 | *GN=aroG* |
| XAC1453 | 1.4543 | 3.45E-11 | *GN=gtlf3b* |
| XAC0636 | 1.4519 | 2.25E-10 | *GN=xerC* |
| XAC3323 | 1.4493 | 2.22E-45 | *GN=col-7* |
| XAC3198 | 1.4474 | 0.0001696 | *GN=ssuA* |
| XAC0465 | 1.4432 | 2.84E-12 | *GN=npr* |
| XAC3325 | 1.4366 | 3.63E-20 | *-//-* |
| XAC3193 | 1.4351 | 1.87E-12 | *-//-* |
| XAC0175 | 1.4325 | 8.60E-07 | *GN=bkdR* |
| XAC0498 | 1.4304 | 1.67E-19 | *-//-* |
| XAC1309 | 1.4285 | 5.00E-07 | *GN=ganB* |
| XAC4354 | 1.4285 | 4.84E-10 | *GN=yhdG* |
| XAC1181 | 1.4251 | 3.53E-08 | *GN=ALKBH2* |
| XAC1141 | 1.4241 | 0.0049877 | *-//-* |
| XAC2709 | 1.4241 | 0.0049877 | *GN=glbN* |
| XAC2611 | 1.4188 | 2.50E-49 | *-//-* |
| XAC2820 | 1.4181 | 2.57E-09 | *-//-* |
| XAC1176 | 1.4156 | 2.24E-06 | *GN=GBA* |
| XAC3153 | 1.4145 | 0.0012964 |  |
| XAC2428 | 1.4139 | 9.58E-14 | *GN=relE3* |
| XAC2066 | 1.4108 | 5.42E-14 | *GN=nolG* |
| XAC1051 | 1.4058 | 3.80E-12 | *-//-* |
| XAC0146 | 1.3979 | 7.08E-05 | *-//-* |
| XAC4042 | 1.3979 | 3.56E-19 | *-//-* |
| XAC3121 | 1.3973 | 0.00081765 | *-//-* |
| XAC2431 | 1.3951 | 0.00094501 |  |
| XAC0438 | 1.3947 | 0.00040294 | *-//-* |
| XAC2515 | 1.3912 | 0.001603 | *GN=PYRAB01370* |
| XAC2626 | 1.3892 | 0.00011421 | *-//-* |
| XAC3001 | 1.3867 | 0.00090818 | *GN=yusP* |
| XAC0684 | 1.3843 | 5.61E-11 | *GN=rcp1* |
| XAC2743 | 1.3821 | 0 | *GN=oar* |
| XAC1300 | 1.381 | 3.14E-25 | *GN=hslR* |
| XAC3662 | 1.3781 | 0.00042973 | *-//-* |
| XAC0182 | 1.3721 | 2.02E-06 | *GN=yehX* |
| XAC2462 | 1.3701 | 7.91E-29 | *GN=ybeZ* |
| XAC1895 | 1.3697 | 2.06E-08 | *GN=tse* |
| XAC0154 | 1.3655 | 6.70E-70 | *GN=glgE* |
| XAC3967 | 1.3627 | 9.88E-07 | *-//-* |
| XAC3071 | 1.361 | 1.20E-17 | *GN=pupA* |
| XAC3962 | 1.3573 | 0.003069 | *-//-* |
| XAC1136 | 1.3522 | 4.14E-06 | *GN=prpR* |
| XAC1285 | 1.3514 | 9.47E-05 | *GN=bglA* |
| XAC2477 | 1.351 | 4.38E-13 | *GN=NGR_a01380* |
| XAC0430 | 1.3504 | 0.0018221 | *-//-* |
| XAC1767 | 1.3499 | 4.45E-09 | *GN=gbpR* |
| XAC2435 | 1.3448 | 0.0010855 | *-//-* |
| XAC3676 | 1.3402 | 9.04E-12 | *GN=PA3106* |
| XAC0015 | 1.3377 | 7.64E-35 | *-//-* |
| XAC2575 | 1.3372 | 4.87E-26 | *-//-* |
| XAC0790 | 1.336 | 0.00028033 | *-//-* |
| XAC3868 | 1.3343 | 6.74E-33 | *GN=yliI* |
| XAC0597 | 1.3301 | 1.10E-08 | *GN=sam* |
| XAC2808 | 1.328 | 6.74E-14 | *-//-* |
| XAC1597 | 1.3263 | 0.00020425 | *-//-* |
| XAC2250 | 1.3198 | 1.44E-20 | *-//-* |
| XAC2491 | 1.3162 | 3.06E-31 | *GN=yjiN* |
| XAC3245 | 1.3142 | 1.43E-36 | *GN=wapA* |
| XAC2893 | 1.3098 | 2.05E-67 | *GN=yagR* |
| XAC3966 | 1.3097 | 1.20E-25 | *-//-* |
| XAC1790 | 1.3095 | 0.00402 | *-//-* |
| XAC0761 | 1.3059 | 2.63E-09 | *-//-* |
| XAC0067 | 1.3037 | 4.41E-33 | *-//-* |
| XAC1278 | 1.2976 | 2.88E-10 | *GN=ybaA* |
| XAC0027 | 1.2967 | 2.31E-25 | *-//-* |
| XAC4082 | 1.2966 | 9.97E-17 | *GN=Rv2402* |
| XAC1789 | 1.291 | 5.95E-08 | *-//-* |
| XAC0043 | 1.2904 | 5.17E-06 | *GN=wcaJ* |
| XAC4052 | 1.2874 | 7.45E-15 | *GN=tonB* |
| XAC2750 | 1.2866 | 3.34E-23 | *GN=gor* |
| XAC3239 | 1.2852 | 1.28E-41 | *GN=pilB* |
| XAC1446 | 1.2833 | 7.61E-40 | *GN=emrB* |
| XAC3875 | 1.2822 | 2.52E-23 | *GN=Daci_4966* |
| XAC1643 | 1.2802 | 4.15E-54 | *-//-* |
| XAC1390 | 1.2796 | 0.0018204 | *GN=yaeR* |
| XAC2731 | 1.2796 | 1.04E-05 | *GN=nudK* |
| XAC3805 | 1.2774 | 4.39E-07 | *GN=pilA* |
| XAC2116 | 1.2747 | 6.32E-06 | *GN=phhB* |
| XAC2997 | 1.2656 | 0.0030874 | *-//-* |
| XAC3005 | 1.2632 | 7.22E-10 | *GN=yjfK* |
| XAC0903 | 1.257 | 3.20E-27 | *GN=rnk* |
| XAC0437 | 1.2546 | 1.42E-10 | *-//-* |
| XAC1784 | 1.2515 | 0.0017617 | *GN=folK* |
| XAC3689 | 1.2476 | 0.0032631 | *GN=lrp* |
| XAC2561 | 1.2475 | 2.90E-05 | *GN=blc* |
| XAC3533 | 1.2472 | 5.03E-132 | *-//-* |
| XAC3874 | 1.241 | 9.81E-06 | *-//-* |
| XAC3228 | 1.24 | 6.93E-34 | *-//-* |
| XAC0089 | 1.2359 | 2.12E-06 | *-//-* |
| XAC3367 | 1.2327 | 7.32E-12 | *GN=BLi01617* |
| XAC3311 | 1.23 | 5.86E-09 | *-//-* |
| XAC2029 | 1.2292 | 0.00011554 | *-//-* |
| XAC0130 | 1.2286 | 0.00021982 | *-//-* |
| XAC3322 | 1.2276 | 1.19E-27 | *-//-* |
| XAC0120 | 1.2249 | 3.86E-23 | *GN=pmbA* |
| XAC0281 | 1.2247 | 3.18E-11 | *GN=ohrR* |
| XAC0926 | 1.2205 | 1.08E-26 | *GN=yitW* |
| XAC0499 | 1.2204 | 5.61E-39 | *GN=erpA* |
| XAC2506 | 1.2193 | 1.26E-06 | *-//-* |
| XAC1399 | 1.2149 | 4.41E-08 | *-//-* |
| XAC2507 | 1.2138 | 1.06E-09 | *-//-* |
| XAC3341 | 1.2119 | 8.18E-11 | *GN=cysK* |
| XAC2083 | 1.2118 | 2.52E-28 | *GN=VEP1* |
| XAC3700 | 1.2116 | 2.45E-13 | *GN=yadG* |
| XAC0026 | 1.2106 | 7.83E-28 | *GN=PTT_17836* |
| XAC0212 | 1.2065 | 2.64E-32 | *-//-* |
| XAC0560 | 1.2052 | 0.00042346 | *-//-* |
| XAC0457 | 1.2022 | 0.00036906 | *GN=mrpG* |
| XAC3983 | 1.2013 | 3.13E-20 | *-//-* |
| XAC1452 | -1.2066 | 9.67E-13 | *-//-* |
| XAC0476 | -1.2098 | 1.49E-20 | *GN=trpE* |
| XAC3823 | -1.2099 | 2.30E-05 | *GN=SCO5481* |
| XAC1030 | -1.2105 | 6.35E-16 | *-//-* |
| XAC2192 | -1.2162 | 3.90E-08 | *GN=fecR* |
| XAC4074 | -1.217 | 3.59E-50 | *GN=nrdB* |
| XAC4000 | -1.2203 | 0.0011727 | *GN=yojE* |
| XAC2585 | -1.2293 | 6.66E-39 |  |
| XAC1854 | -1.2296 | 0.00040073 | *-//-* |
| XAC2144 | -1.2359 | 0.00013556 |  |
| XAC0195 | -1.2383 | 1.13E-24 | *GN=jhp_0176* |
| XAC0950 | -1.239 | 5.25E-36 | *GN=prs* |
| XAC4085 | -1.2416 | 8.80E-11 | *GN=XAC4085* |
| XAC1208 | -1.2422 | 0.00077022 | *-//-* |
| XAC3475 | -1.2426 | 0.0012943 | *-//-* |
| XAC3109 | -1.2513 | 4.31E-16 | *GN=mrcB* |
| XAC4064 | -1.2516 | 0.00018139 | *GN=cdhR* |
| XAC0264 | -1.256 | 1.14E-35 | *GN=Mccc2* |
| XAC1899 | -1.257 | 0.0029475 | *GN=tsr* |
| XAC1263 | -1.2579 | 3.99E-21 | *GN=radA* |
| XAC0198 | -1.2616 | 1.45E-08 | *GN=yheT* |
| XAC2729 | -1.2673 | 7.25E-11 | *GN=mltD* |
| XAC1219 | -1.2718 | 4.50E-07 | *-//-* |
| XAC2112 | -1.2724 | 1.74E-09 | *GN=rluC* |
| XAC2989 | -1.2739 | 0.0031817 | *GN=plaP* |
| XAC3872 | -1.2772 | 3.88E-98 | *GN=rpsU* |
| XAC3809 | -1.2825 | 4.21E-10 | *-//-* |
| XAC1482 | -1.2836 | 1.50E-08 | *GN=bepF* |
| XAC3816 | -1.2931 | 1.57E-05 | *-//-* |
| XAC1705 | -1.296 | 7.63E-06 | *GN=yyaJ* |
| XAC3474 | -1.2964 | 5.18E-06 | *GN=citH* |
| XAC2813 | -1.299 | 1.66E-38 | *GN=deaD* |
| XAC0492 | -1.302 | 4.79E-07 | *-//-* |
| XAC1484 | -1.3023 | 0.00015082 |  |
| XAC1483 | -1.3027 | 4.33E-14 | *GN=bepE* |
| XAC1760 | -1.3049 | 1.30E-27 | *GN=dapA* |
| XAC3532 | -1.3059 | 6.99E-06 | *GN=phnR* |
| XAC1969 | -1.3085 | 4.57E-35 | *GN=rpoN* |
| XAC1814 | -1.3132 | 0.00452 | *GN=hpmB* |
| XAC0207 | -1.3174 | 6.64E-06 | *GN=ntrB* |
| XAC3712 | -1.3209 | 2.37E-23 | *GN=Ecel1* |
| XAC1761 | -1.3233 | 2.39E-47 | *-//-* |
| XAC1397 | -1.3266 | 3.82E-13 | *-//-* |
| XAC0300 | -1.3298 | 0.00031287 | *GN=pucG* |
| XAC3959 | -1.3303 | 4.25E-135 | *GN=desA3* |
| XAC0479 | -1.3325 | 3.56E-05 | *GN=aq_928* |
| XAC3441 | -1.3476 | 0.0010241 | *-//-* |
| XAC1466 | -1.3491 | 1.53E-37 | *-//-* |
| XAC4093 | -1.3508 | 7.77E-10 | *-//-* |
| XAC1426 | -1.3571 | 3.89E-09 | *-//-* |
| XAC1427 | -1.362 | 3.29E-14 | *GN=pru* |
| XAC1041 | -1.371 | 6.13E-05 | *GN=phoR* |
| XAC2584 | -1.3712 | 2.60E-93 | *GN=exoP* |
| sRNA00065 | -1.3964 | 2.19E-08 | *-//-* |
| XAC3213 | -1.4002 | 0.0040981 | *GN=tsr* |
| XAC4342 | -1.4012 | 2.97E-49 | *GN=ttg2D* |
| XAC3376 | -1.4072 | 5.38E-08 | *GN=BB_0173* |
| XAC0656 | -1.408 | 2.68E-42 | *GN=mreB* |
| XAC3374 | -1.4094 | 1.89E-15 | *-//-* |
| XAC0474 | -1.41 | 3.50E-11 | *GN=ats1* |
| Novel00039 | -1.413 | 3.33E-11 | *-//-* |
| XAC1637 | -1.4138 | 2.94E-07 | *GN=hutH* |
| XAC3086 | -1.4162 | 0.0045823 | *-//-* |
| XAC3736 | -1.4269 | 2.45E-61 | *GN=cydB* |
| XAC1993 | -1.4287 | 6.40E-08 | *GN=PA1727* |
| XAC0484 | -1.4327 | 6.20E-113 | *GN=speD* |
| XAC4344 | -1.4378 | 7.36E-25 | *GN=mlaA* |
| XAC0659 | -1.4465 | 1.80E-12 | *GN=mrdA* |
| XAC3057 | -1.4493 | 1.42E-53 | *GN=flp* |
| XAC3337 | -1.4524 | 3.21E-05 | *-//-* |
| XAC2823 | -1.458 | 1.89E-05 | *GN=ogt* |
| XAC1503 | -1.4727 | 1.94E-69 | *-//-* |
| XAC4343 | -1.4755 | 2.29E-07 | *-//-* |
| XAC0480 | -1.4776 | 5.34E-10 | *GN=trpD* |
| XAC0296 | -1.4826 | 3.78E-05 | *-//-* |
| XAC2531 | -1.4829 | 2.72E-125 | *GN=btuB* |
| XAC1519 | -1.4953 | 4.55E-43 | *GN=recN* |
| XAC3988 | -1.4955 | 8.68E-08 | *-//-* |
| XAC2571 | -1.5011 | 6.83E-05 | *GN=fabH* |
| XAC2395 | -1.5238 | 0 | *GN=cspA* |
| XAC2338 | -1.5262 | 2.95E-08 | *-//-* |
| XAC2008 | -1.5279 | 1.99E-35 | *GN=lolA* |
| XAC3605 | -1.5366 | 1.24E-26 | *-//-* |
| sRNA00024 | -1.5502 | 1.27E-11 | *-//-* |
| XAC1953 | -1.5637 | 1.43E-08 | *GN=fliG* |
| XAC0260 | -1.5679 | 0.00062509 | *-//-* |
| XAC0215 | -1.5745 | 3.37E-15 | *-//-* |
| XAC4340 | -1.5775 | 9.06E-28 | *GN=mlaE* |
| XAC1414 | -1.5813 | 2.87E-26 | *GN=XF_1047* |
| XAC1950 | -1.583 | 0.00087392 | *-//-* |
| XAC0176 | -1.59 | 1.66E-05 | *GN=fpvA* |
| XAC3377 | -1.5995 | 2.76E-07 | *-//-* |
| XAC1894 | -1.6016 | 9.78E-05 | *GN=tsr* |
| XAC2962 | -1.6017 | 1.28E-19 | *-//-* |
| XAC3831 | -1.603 | 4.36E-79 | *GN=rho* |
| XAC4025 | -1.6068 | 4.31E-18 | *GN=dusA* |
| XAC2988 | -1.6098 | 9.64E-10 | *GN=ahyR* |
| XAC1796 | -1.6131 | 4.02E-06 | *GN=manA* |
| XAC1589 | -1.6156 | 2.38E-24 | *-//-* |
| XAC2081 | -1.6173 | 3.47E-19 | *GN=lolC* |
| XAC1088 | -1.6194 | 1.33E-23 | *-//-* |
| XAC3378 | -1.6194 | 2.36E-18 | *GN=Mb1516* |
| XAC3218 | -1.6222 | 1.57E-38 | *GN=bamD* |
| XAC3145 | -1.6263 | 9.53E-35 | *GN=tolQ* |
| XAC0477 | -1.6432 | 1.53E-23 | *GN=ltaE* |
| XAC0478 | -1.6443 | 4.40E-18 | *GN=trpG* |
| XAC2866 | -1.6455 | 9.13E-05 | *GN=tcp* |
| XAC0299 | -1.6516 | 2.96E-05 | *GN=cda1* |
| XAC3529 | -1.664 | 7.16E-07 | *GN=fatA* |
| XAC3143 | -1.6666 | 6.45E-19 | *-//-* |
| XAC2773 | -1.6703 | 1.44E-38 | *GN=oar* |
| XAC1042 | -1.671 | 2.13E-10 | *GN=phoB* |
| XAC2036 | -1.6777 | 3.50E-62 | *GN=mntH* |
| XAC0415 | -1.6897 | 5.01E-07 | *GN=hrpA1* |
| XAC2822 | -1.6901 | 2.53E-19 | *GN=alkA* |
| XAC1424 | -1.6914 | 0.00013827 | *-//-* |
| XAC4278 | -1.6922 | 1.43E-36 | *GN=XAC4278* |
| XAC1636 | -1.694 | 2.12E-05 | *-//-* |
| XAC3686 | -1.6972 | 1.36E-37 | *-//-* |
| XAC0435 | -1.6975 | 1.95E-07 |  |
| XAC1975 | -1.699 | 1.03E-45 | *GN=fliC* |
| XAC0204 | -1.7025 | 2.92E-71 | *GN=glnA* |
| XAC3512 | -1.7168 | 3.10E-11 | *GN=arsC* |
| XAC4219 | -1.7209 | 8.71E-41 | *GN=LSB3* |
| XAC1240 | -1.7535 | 1.33E-23 | *-//-* |
| XAC4021 | -1.754 | 5.97E-08 | *-//-* |
| XAC3604 | -1.7563 | 0 | *-//-* |
| XAC2245 | -1.7604 | 1.68E-16 | *-//-* |
| XAC1944 | -1.7605 | 3.91E-09 | *GN=fliP* |
| XAC1540 | -1.7628 | 1.82E-05 | *-//-* |
| XAC0394 | -1.7728 | 1.37E-08 | *GN=nopX* |
| XAC0301 | -1.7789 | 5.88E-08 | *GN=amaB* |
| XAC2335 | -1.7819 | 8.61E-211 | *GN=cydD* |
| XAC0286 | -1.7858 | 1.61E-17 | *-//-* |
| XAC1425 | -1.7871 | 1.24E-18 |  |
| XAC1496 | -1.7901 | 1.42E-47 | *-//-* |
| XAC2963 | -1.804 | 1.97E-75 | *GN=GLU-D1-2B* |
| XAC0076 | -1.8173 | 2.94E-08 | *GN=GDE1* |
| XAC1743 | -1.8239 | 6.27E-107 | *GN=csrA* |
| XAC3225 | -1.8242 | 1.77E-103 | *GN=mltB* |
| XAC1938 | -1.8277 | 1.47E-05 | *GN=PA1727* |
| XAC3890 | -1.8308 | 0 | *GN=putA* |
| XAC4096 | -1.8314 | 5.77E-18 | *-//-* |
| XAC2082 | -1.8408 | 4.22E-22 | *GN=lolD* |
| XAC2334 | -1.8431 | 2.50E-176 | *GN=cydC* |
| XAC0077 | -1.8508 | 1.01E-08 | *GN=yprB* |
| XAC1635 | -1.8604 | 5.34E-08 | *GN=hutU* |
| XAC2868 | -1.8637 | 0.0025566 | *GN=dosP* |
| XAC3132 | -1.871 | 2.76E-25 | *GN=tsr* |
| XAC1489 | -1.8759 | 8.65E-16 | *-//-* |
| XAC4020 | -1.8771 | 6.03E-09 | *-//-* |
| XAC3146 | -1.8791 | 1.13E-21 | *GN=HI_0386* |
| XAC4036 | -1.8867 | 2.57E-15 | *GN=cysJ* |
| XAC2897 | -1.9128 | 9.89E-07 | *GN=cph2* |
| XAC2406 | -1.9171 | 4.30E-68 | *GN=amiC* |
| XAC0623 | -1.9187 | 1.95E-19 | *GN=ydiY* |
| XAC0501 | -1.9208 | 2.60E-14 | *-//-* |
| XAC2865 | -1.9356 | 1.93E-05 | *GN=cheA* |
| XAC0940 | -1.9417 | 1.21E-05 | *-//-* |
| XAC0398 | -1.9497 | 0.00079523 | *-//-* |
| XAC1892 | -1.9605 | 2.22E-05 | *GN=tsr* |
| XAC2967 | -1.9741 | 9.40E-45 | *GN=kdsD* |
| XAC2054 | -1.9761 | 4.33E-45 | *GN=NGR_a02630* |
| XAC2755 | -1.9839 | 1.86E-35 | *GN=ydhO* |
| XAC2113 | -1.9864 | 1.31E-16 | *GN=yacH* |
| XAC3176 | -1.9882 | 1.08E-06 | *GN=fecA* |
| XAC3230 | -1.9905 | 4.78E-27 | *GN=MAV_4644* |
| XAC0823 | -1.9929 | 1.42E-05 | *GN=hgbC* |
| XAC3994 | -2.0026 | 2.66E-09 | *GN=desK* |
| XAC4030 | -2.024 | 1.02E-05 | *GN=katB* |
| XAC0405 | -2.029 | 1.14E-12 | *GN=hrpC2* |
| XAC0271 | -2.0371 | 0.0021715 | *-//-* |
| XAC0270 | -2.0393 | 3.48E-10 | *-//-* |
| XAC3666 | -2.047 | 1.39E-07 | *-//-* |
| XAC4033 | -2.0524 | 1.24E-08 | *-//-* |
| XAC0029 | -2.0613 | 2.27E-54 | *GN=egl* |
| XAC1435 | -2.0667 | 1.11E-08 | *GN=foxA* |
| XAC1226 | -2.0714 | 7.92E-92 | *GN=minC* |
| XAC1855 | -2.0782 | 2.66E-46 | *GN=feoB* |
| XAC2976 | -2.0786 | 7.73E-49 | *GN=XAC2976* |
| XAC1578 | -2.0963 | 6.80E-05 | *GN=pstS* |
| XAC1932 | -2.0968 | 3.54E-08 | *GN=cheY* |
| XAC4326 | -2.1053 | 1.35E-16 | *GN=DUR1,2* |
| XAC1982 | -2.1116 | 1.26E-08 | *GN=flgF* |
| Novel00032 | -2.1151 | 1.23E-31 | *-//-* |
| XAC2388 | -2.1163 | 1.59E-07 | *-//-* |
| XAC4007 | -2.1203 | 3.77E-74 | *GN=XAC4007* |
| XAC2942 | -2.1235 | 0.002796 | *GN=XAC2942* |
| XAC4213 | -2.1337 | 4.13E-45 | *GN=MUC5AC* |
| XAC1931 | -2.147 | 6.31E-09 | *GN=cheZ* |
| XAC3646 | -2.1846 | 5.01E-07 | *-//-* |
| Novel00035 | -2.2092 | 1.47E-27 | *-//-* |
| XAC0421 | -2.2174 | 3.79E-14 | *GN=opgB* |
| XAC0999 | -2.2196 | 2.33E-05 | *GN=cirA* |
| XAC1941 | -2.2199 | 0.0019239 | *GN=fliR* |
| XAC4199 | -2.2298 | 4.70E-14 | *GN=pvaA* |
| XAC1940 | -2.2302 | 9.73E-10 | *GN=CC_0091* |
| XAC0217 | -2.2389 | 1.40E-06 | *GN=AGAP012208* |
| Novel00043 | -2.252 | 9.88E-155 | *-//-* |
| XAC3528 | -2.2543 | 1.93E-07 | *-//-* |
| XAC1951 | -2.256 | 1.43E-17 | *GN=fliI* |
| XAC1949 | -2.264 | 1.97E-26 | *-//-* |
| XAC0552 | -2.2734 | 2.07E-15 | *GN=pcp* |
| XAC1171 | -2.2773 | 1.13E-05 | *GN=fnkB* |
| XAC1577 | -2.2777 | 5.96E-29 | *GN=pstS* |
| XAC2786 | -2.279 | 1.69E-23 | *-//-* |
| XAC1952 | -2.2921 | 7.37E-09 | *-//-* |
| XAC0272 | -2.3095 | 6.11E-15 | *-//-* |
| XAC2009 | -2.3454 | 2.00E-19 | *GN=Srrm2* |
| XAC0754 | -2.3505 | 1.60E-07 | *-//-* |
| XAC1856 | -2.3516 | 5.00E-08 | *-//-* |
| XAC1934 | -2.3675 | 3.02E-10 | *GN=ylxH* |
| XAC1930 | -2.3919 | 9.80E-19 | *GN=cheA* |
| XAC2941 | -2.3999 | 3.60E-12 | *GN=bfrD* |
| sRNA00050 | -2.4355 | 1.32E-56 | *-//-* |
| XAC0400 | -2.458 | 1.82E-05 | *-//-* |
| XAC3115 | -2.4846 | 6.14E-25 | *GN=pqqC* |
| XAC2387 | -2.505 | 3.20E-09 | *GN=rnaSA3* |
| XAC2922 | -2.5152 | 2.64E-56 | *GN=pelC* |
| XAC2411 | -2.5491 | 8.38E-21 | *GN=fp-1* |
| XAC0140 | -2.5615 | 3.03E-144 | *GN=cadC* |
| XAC1749 | -2.5727 | 3.24E-84 | *GN=med15* |
| XAC2931 | -2.581 | 2.77E-26 | *-//-* |
| Novel00045 | -2.6096 | 9.01E-23 | *GN=DUR1,2* |
| XAC0277 | -2.6134 | 4.79E-25 | *-//-* |
| XAC0337 | -2.6424 | 1.79E-06 | *GN=kdgT* |
| XAC1412 | -2.671 | 9.50E-07 | *-//-* |
| XAC1576 | -2.671 | 1.53E-09 | *GN=pstC* |
| XAC1933 | -2.6854 | 7.24E-12 | *GN=fliA* |
| XAC0607 | -2.6961 | 1.85E-09 | *-//-* |
| XAC0880 | -2.7035 | 6.96E-14 | *GN=pcaQ* |
| XAC1795 | -2.7235 | 3.10E-11 | *GN=PA1727* |
| XAC2447 | -2.7359 | 0.00014684 | *GN=cheW* |
| XAC0338 | -2.7471 | 1.68E-14 | *-//-* |
| XAC1983 | -2.751 | 2.42E-22 | *GN=flgE* |
| XAC1434 | -2.7518 | 6.13E-74 | *-//-* |
| XAC1792 | -2.7523 | 3.43E-09 | *-//-* |
| XAC2821 | -2.7639 | 2.36E-39 | *-//-* |
| XAC1954 | -2.7658 | 7.29E-20 | *GN=fliF* |
| XAC1172 | -2.7673 | 3.36E-33 | *-//-* |
| XAC1942 | -2.7865 | 0.00038265 | *-//-* |
| XAC0543 | -2.7888 | 7.16E-51 | *-//-* |
| XAC0879 | -2.7939 | 0.0048125 | *GN=galA* |
| XAC1574 | -2.7942 | 1.46E-11 | *GN=pstB* |
| XAC3878 | -2.7953 | 1.66E-36 | *GN=dsbD1* |
| XAC1573 | -2.8154 | 7.00E-13 | *GN=phoU* |
| XAC1976 | -2.816 | 7.13E-19 | *GN=flgL* |
| XAC0399 | -2.8264 | 8.94E-17 | *-//-* |
| XAC1575 | -2.832 | 2.61E-07 | *GN=pstA* |
| XAC1834 | -2.8393 | 1.00E-17 | *GN=hisF* |
| XAC0414 | -2.845 | 2.94E-06 | *GN=yscT* |
| Novel00037 | -2.8455 | 5.04E-29 | *-//-* |
| XAC1988 | -2.8726 | 1.54E-06 | *-//-* |
| XAC1833 | -2.906 | 6.72E-21 | *GN=hisA* |
| XAC2448 | -2.908 | 2.51E-16 | *GN=tsr* |
| XAC1948 | -2.9248 | 1.76E-08 | *GN=fliL* |
| XAC3439 | -2.9294 | 1.01E-29 | *-//-* |
| XAC3880 | -2.9394 | 0 | *-//-* |
| XAC1980 | -2.9579 | 1.89E-08 | *GN=flgH* |
| XAC1936 | -2.9695 | 6.93E-18 | *GN=flhA* |
| XAC1835 | -2.9827 | 1.12E-21 | *GN=hisI* |
| XAC4327 | -2.9834 | 2.89E-20 | *GN=atzF* |
| XAC0406 | -2.9876 | 7.26E-12 | *GN=hrcU* |
| XAC2654 | -2.9891 | 2.14E-37 | *GN=EGC2* |
| XAC0410 | -3.0255 | 1.95E-12 | *-//-* |
| XAC3848 | -3.0428 | 0 | *GN=mdtA* |
| XAC1977 | -3.0454 | 1.74E-23 | *GN=flgK* |
| XAC1937 | -3.0715 | 2.47E-05 | *GN=flhB* |
| XAC0412 | -3.0784 | 6.51E-09 | *GN=hrpB6* |
| XAC3114 | -3.0797 | 0 | *GN=pqqB* |
| sRNA00035 | -3.0881 | 1.54E-29 | *-//-* |
| XAC0878 | -3.1144 | 7.74E-12 | *GN=ligB* |
| XAC1984 | -3.135 | 3.25E-11 | *GN=flgD* |
| XAC1265 | -3.1667 | 1.49E-16 | *GN=chvI* |
| XAC1978 | -3.1714 | 9.31E-16 | *GN=flgJ* |
| XAC1985 | -3.1884 | 0.00022422 | *GN=flgC* |
| XAC4024 | -3.1931 | 3.09E-104 | *-//-* |
| XAC3085 | -3.1966 | 2.69E-33 | *-//-* |
| XAC0396 | -3.2063 | 6.33E-14 | *-//-* |
| XAC0822 | -3.2279 | 5.15E-29 | *-//-* |
| XAC3117 | -3.2318 | 9.23E-164 | *GN=pqqE* |
| XAC3446 | -3.263 | 2.16E-108 | *GN=tolB* |
| XAC1266 | -3.2775 | 1.50E-52 | *GN=hrpB* |
| XAC3125 | -3.2938 | 9.37E-54 | *-//-* |
| XAC3370 | -3.3359 | 1.12E-50 | *GN=fhuE* |
| XAC1832 | -3.343 | 1.19E-13 | *GN=hisH* |
| XAC1831 | -3.3447 | 4.69E-71 | *GN=hisB* |
| XAC3647 | -3.3791 | 2.37E-29 | *GN=aroQ* |
| XAC1947 | -3.492 | 1.35E-08 | *GN=fliM* |
| XAC1829 | -3.4986 | 8.71E-61 | *GN=hisD* |
| XAC1830 | -3.506 | 5.38E-41 | *GN=hisC* |
| XAC1945 | -3.5139 | 1.58E-06 | *-//-* |
| sRNA00052 | -3.5222 | 0 | *-//-* |
| XAC1827 | -3.53 | 7.49E-10 | *-//-* |
| XAC2914 | -3.5366 | 4.33E-139 | *-//-* |
| XAC0223 | -3.5898 | 0 | *-//-* |
| XAC0028 | -3.619 | 1.35E-31 | *GN=egl* |
| XAC1979 | -3.7131 | 1.32E-14 | *GN=flgI* |
| XAC1981 | -3.7208 | 9.54E-24 | *GN=flgG* |
| XAC1986 | -3.7208 | 1.21E-12 | *GN=flgB* |
| XAC1828 | -3.7577 | 1.05E-43 | *GN=hisG* |
| XAC0413 | -3.7641 | 0.00020245 | *-//-* |
| XAC4333 | -3.8438 | 1.72E-19 | *-//-* |
| XAC0401 | -3.8586 | 0.002682 | *-//-* |
| XAC2951 | -3.9193 | 0 | *GN=HI_1008* |
| XAC1946 | -3.9225 | 0.00085338 | *GN=fliN* |
| XAC0408 | -4.0202 | 3.45E-12 | *-//-* |
| XAC0411 | -4.0469 | 6.46E-07 | *-//-* |
| XAC2853 | -4.0975 | 1.18E-20 | *GN=MIMI_L477* |
| XAC0407 | -4.2062 | 8.22E-28 | *-//-* |
| XAC0404 | -4.256 | 2.06E-16 | *GN=hpaP* |
| XAC1955 | -4.3033 | 2.79E-06 | *GN=fliE* |
| Novel00031 | -4.3235 | 0 | *GN=HI_1008* |
| XAC0403 | -4.3939 | 1.11E-17 | *GN=yscQ* |
| XAC1935 | -4.4217 | 6.82E-24 | *GN=flhF* |
| XAC3155 | -4.4261 | 1.48E-60 | *-//-* |
| XAC0409 | -4.4763 | 6.59E-20 | *GN=hrpB3* |
| XAC1113 | -4.6118 | 2.20E-229 | *GN=HI_0389* |
| XAC0742 | -4.6309 | 1.93E-98 | *-//-* |
| XAC4026 | -4.7178 | 1.73E-30 | *-//-* |
| XAC2353 | -4.8316 | 1.05E-93 | *-//-* |
| XAC1008 | -4.8445 | 6.03E-35 | *-//-* |
| XAC1651 | -5.1217 | 6.41E-61 | *GN=tonB* |
| XAC3856 | -5.1384 | 5.05E-92 | *-//-* |
| XAC0397 | -5.1637 | 0 | *-//-* |
| XAC1471 | -5.557 | 0 | *GN=ycfJ* |
| Novel00021 | -5.8261 | 7.53E-254 | *-//-* |
| XAC0416 | -6.0633 | 3.18E-183 | *-//-* |
| XAC3445 | -6.2088 | 5.00E-102 | *GN=aarP* |
| XAC3444 | -6.5257 | 0 | *GN=btuB* |
| XAC3664 | -7.8484 | 0 | *GN=ompW* |
| XAC4023 | -8.1681 | 3.33E-252 | *GN=phoP* |

Note: - , do not show in the RNA-Seq; log_2_.Fold_change: log_2_(read count_△*phoP* / read count_XHG3)
