# Supplementary material for: Global Regulator PhoP is Necessary for Motility, Biofilm Formation, Exoenzyme Production, and Virulence of Xanthomonas citri Subsp. citri on Citrus Plants
Source: Genes (Basel). 2019 May 6;10(5):340. doi: 10.3390/genes10050340 (PMC6562643; doi:10.3390/genes10050340)
Supplement: Supplementary file 1 [file genes-10-00340-s001.zip › Fig and Supplementary/Table S4.docx]

**Table S4. Analysis of KEGG pathway significant enrichment between △*phoP* and XHG3**

| Term | Id | Sample number | Background number | P-Value | KO_name |
| --- | --- | --- | --- | --- | --- |
| Flagellar assembly | XAC02040 | 28 | 35 | 0 | fliC\|flgL\|flgK\|fliF\|fliE\|fliH\|flgI\|flhA\|flhB\|flgA\|fliP\|flgC\|flgD\|flgB\|flgG\|flgH\|flgE\|flgF\|fleN\|fliG\|fliR\|fliQ\|fliO\|fliJ\|fliM\|fliN\|fliK\|fliI\| |
| Two-component system | XAC02020 | 45 | 121 | 5.54E-09 | None\|pilJ\|pilL\|bla\|cioA\|mcp\|dctA\| \|None\| \|None\|fliC\|tsr\|tsr\|tsr\|cheA\|cheY\|fliA\| \|tsr\|None\|None\|None\|None\|None\|None\|None\|mcp\|cioB\|csrA\|None\|phoR\|phoB\|cheW\|pilG\|pilH\|pilI\|None\|None\|None\|pilA\|None\|None\|rpoN\|glnA\| |
| Histidine metabolism | XAC00340 | 13 | 21 | 2.22E-06 | hisB\|hisC\|hisA\|hisH\|hisI\|hisF\|hutU\|None\|None\|hutH\|hutG\|hisG\|hisD\| |
| Bacterial chemotaxis | XAC02030 | 17 | 48 | 0.000719722 | None\|None\|None\|None\|mcp\|tsr\|tsr\|cheW\|fliM\|fliN\|cheA\|cheZ\|cheY\|mcp\|fliG\|tsr\|tsr\| |
| ABC transporters | XAC02010 | 19 | 62 | 0.00259164 | None\|None\| \| \|None\|lolC\|yadG\|cysW\|ycfV\|None\|None\|None\|None\|None\| \| \|cydC\|strW\|cysU\| |
| Bacterial secretion system | XAC03070 | 20 | 76 | 0.013180816 | virB3\| \|None\|virB2\|None\|None\|virB4\|None\|virB11\|None\|None\|virB9\|virB8\|None\|virD4\|virB1\|None\|None\|virB6\|virB10\| |
| Starch and sucrose metabolism | XAC00500 | 12 | 48 | 0.069220685 | None\|None\|None\|None\|None\| \|None\|None\|galU\|None\|None\| |
| Polycyclic aromatic hydrocarbon degradation | XAC00624 | 3 | 7 | 0.085844351 | pcaH\|None\|ligA\| |
| Alanine, aspartate and glutamate metabolism | XAC00250 | 7 | 25 | 0.090159174 | None\|putA\|glnA\|gabD\|carA\|None\|None\| |
| Phenylalanine, tyrosine and tryptophan biosynthesis | XAC00400 | 7 | 26 | 0.10726341 | hisC\|trpF\|pheA\|None\|None\|None\|None\| |
| Sphingolipid metabolism | XAC00600 | 2 | 5 | 0.182597231 | None\|None\| |
| Other glycan degradation | XAC00511 | 3 | 10 | 0.205027985 | None\| \|None\| |
| Arginine and proline metabolism | XAC00330 | 8 | 36 | 0.205381201 | uahA\|None\|uahA\|uahA\|putA\|glnA\|None\|bioA\|None\| |
| Glycerolipid metabolism | XAC00561 | 3 | 11 | 0.250586437 | None\|None\|None\| |
| Ascorbate and aldarate metabolism | XAC00053 | 3 | 11 | 0.250586437 | None\|None\|None\| |
| Valine, leucine and isoleucine degradation | XAC00280 | 5 | 22 | 0.267203875 | \|None\|None\|None\|ipsI\| |
| Base excision repair | XAC03410 | 4 | 17 | 0.282100329 | xthA2\|None\|None\|None\| |
| Purine metabolism | XAC00230 | 12 | 63 | 0.297838323 | None\|None\|None\|None\|None\|None\|None\|None\|None\|None\|None\|None\| |
| Sulfur metabolism | XAC00920 | 3 | 14 | 0.391577009 | None\|metA\|None\| |
| RNA degradation | XAC03018 | 3 | 15 | 0.437608774 | None\|None\|rho\| |
| Galactose metabolism | XAC00052 | 4 | 21 | 0.437642271 | galU\|dgoK\| \|None\| |
| Tyrosine metabolism | XAC00350 | 3 | 16 | 0.482256765 | hisC\|None\|gabD\| |
| Nucleotide excision repair | XAC03420 | 2 | 10 | 0.490743203 | None\|None\| |
| Cyanoamino acid metabolism | XAC00460 | 2 | 10 | 0.490743203 | \|None\| |
| Benzoate degradation | XAC00362 | 3 | 17 | 0.52514743 | pcaH\|ligA\|None\| |
| Glycerophospholipid metabolism | XAC00564 | 4 | 24 | 0.548013862 | None\|None\|cls\|None\| |
| Novobiocin biosynthesis | XAC00401 | 1 | 5 | 0.581027239 | hisC\| |
| Penicillin and cephalosporin biosynthesis | XAC00311 | 1 | 5 | 0.581027239 | bla\| |
| Taurine and hypotaurine metabolism | XAC00430 | 1 | 5 | 0.581027239 | tauD, ssiD\| |
| Phosphotransferase system (PTS) | XAC02060 | 1 | 5 | 0.581027239 | None\| |
| Terpenoid backbone biosynthesis | XAC00900 | 2 | 12 | 0.593452495 | None\|None\| |
| Synthesis and degradation of ketone bodies | XAC00072 | 1 | 6 | 0.648001615 | ipsI\| |
| Limonene and pinene degradation | XAC00903 | 1 | 6 | 0.648001615 | None\| |
| Styrene degradation | XAC00643 | 1 | 6 | 0.648001615 | None\| |
| Microbial metabolism in diverse environments | XAC01120 | 29 | 191 | 0.650703485 | None\|None\|None\|None\|None\|None\|None\|None\|None\|None\|uahA\|None\|None\|None\|dapA\|glnA\|gabD\|pgmA\|None\|None\|None\|None\|pcaH\|ligA\|None\|rpfA\|None\|None\|None\| |
| Glyoxylate and dicarboxylate metabolism | XAC00630 | 4 | 28 | 0.675332907 | None\|None\|glnA\|rpfA\| |
| Glutathione metabolism | XAC00480 | 4 | 29 | 0.702957948 | gstA\|gst\|None\|None\| |
| Geraniol degradation | XAC00281 | 1 | 7 | 0.704288591 | None\| |
| Biosynthesis of secondary metabolites | XAC01110 | 41 | 274 | 0.707834997 | \| \|None\|hisI\|None\|trpF\|bla\|pheA\|None\|gcd\|None\| \|None\|hisB\|hisC\|hisA\|hisH\|None\|hisF\|galU\|hisD\|None\|None\|None\|None\|dapA\|pgmA\|None\|None\|None\|None\|hisG\|None\|None\|putA\|None\|None\|None\|rpfA\|None\|None\| |
| Pentose and glucuronate interconversions | XAC00040 | 3 | 23 | 0.735345745 | galU\|pel\|None\| |
| Pyrimidine metabolism | XAC00240 | 5 | 40 | 0.788798835 | None\|carA\|pyrC\|None\|None\| |
| Glycine, serine and threonine metabolism | XAC00260 | 4 | 33 | 0.796327277 | None\|betB\|None\|pgmA\| |
| Peptidoglycan biosynthesis | XAC00550 | 2 | 18 | 0.807624953 | None\|ponB\| |
| Pentose phosphate pathway | XAC00030 | 3 | 26 | 0.809031474 | gcd\|None\|None\| |
| Methane metabolism | XAC00680 | 3 | 26 | 0.809031474 | None\|None\|pgmA\| |
| Butanoate metabolism | XAC00650 | 3 | 26 | 0.809031474 | ipsI\|gabD\|None\| |
| beta-Alanine metabolism | XAC00410 | 1 | 10 | 0.824739003 | None\| |
| Lysine degradation | XAC00310 | 1 | 10 | 0.824739003 | None\| |
| Aminobenzoate degradation | XAC00627 | 2 | 19 | 0.831506205 | pcaH\|ligA\| |
| Tryptophan metabolism | XAC00380 | 2 | 20 | 0.852682598 | None\|None\| |
| Ubiquinone and other terpenoid-quinone biosynthesis | XAC00130 | 1 | 11 | 0.852801765 | None\| |
| Selenocompound metabolism | XAC00450 | 1 | 11 | 0.852801765 | None\| |
| Chloroalkane and chloroalkene degradation | XAC00625 | 1 | 11 | 0.852801765 | None\| |
| Cysteine and methionine metabolism | XAC00270 | 3 | 29 | 0.864627122 | None\|metA\|None\| |
| Amino sugar and nucleotide sugar metabolism | XAC00520 | 3 | 30 | 0.879713838 | galU\| \|None\| |
| Nitrogen metabolism | XAC00910 | 4 | 39 | 0.890608626 | None\|glnA\|None\|None\| |
| Pyruvate metabolism | XAC00620 | 3 | 31 | 0.893288849 | gloA\|None\|None\| |
| Phenylalanine metabolism | XAC00360 | 1 | 13 | 0.896186338 | hisC\| |
| Homologous recombination | XAC03440 | 2 | 23 | 0.902452712 | None\|recA\| |
| Lysine biosynthesis | XAC00300 | 1 | 14 | 0.912825582 | dapA\| |
| Oxidative phosphorylation | XAC00190 | 5 | 50 | 0.919840706 | cioA\|cioB\|atpH, uncH\| \|None\| |
| Fatty acid metabolism | XAC00071 | 1 | 15 | 0.926802547 | None\| |
| Folate biosynthesis | XAC00790 | 1 | 15 | 0.926802547 | folK\| |
| DNA replication | XAC03030 | 1 | 15 | 0.926802547 | None\| |
| Glycolysis / Gluconeogenesis | XAC00010 | 3 | 35 | 0.934848281 | None\|None\|pgmA\| |
| Porphyrin and chlorophyll metabolism | XAC00860 | 2 | 27 | 0.944712694 | \|None\| |
| Metabolic pathways | XAC01100 | 87 | 623 | 0.945295967 | None\|None\|None\|pheA\|None\|None\| \|None\|None\|dapA\|dgoK\|glnA\|None\|None\|None\|None\|None\|None\|None\|rpfA\|None\|None\| \|trpF\|None\|hisB\|hisC\|hisA\|hisH\|hisI\| \|None\|fabH\|None\|gabD\|None\|hisG\|hisD\|None\|None\|None\|atpH, uncH\|None\|None\| \|pyrC\|None\|None\|cls\|None\|None\|uahA\|uahA\|None\|folK\|betB\|None\|None\|None\|None\|None\|cioB\|None\|None\|None\|None\|hisF\|gcd\|None\|hutH\|hutG\|hutU\|galU\|None\|uahA\|None\|None\|None\|putA\|None\|None\|pgmA\|carA\|None\|None\|cioA\|metA\|None\| |
| Fructose and mannose metabolism | XAC00051 | 1 | 17 | 0.948402719 | \| |
| Propanoate metabolism | XAC00640 | 1 | 19 | 0.963638073 | None\| |
| Fatty acid biosynthesis | XAC00061 | 1 | 20 | 0.969477835 | fabH\| |
| Ribosome | XAC03010 | 5 | 60 | 0.973267858 | rpsA\|rpsU\|rpmJ\|rpsS\|rplQ\| |
| Citrate cycle (TCA cycle) | XAC00020 | 1 | 24 | 0.984857019 | rpfA\| |
