# Supplementary figures and images for: Global Regulator PhoP is Necessary for Motility, Biofilm Formation, Exoenzyme Production, and Virulence of Xanthomonas citri Subsp. citri on Citrus Plants
Source: Genes (Basel). 2019 May 6;10(5):340. doi: 10.3390/genes10050340 (PMC6562643; doi:10.3390/genes10050340)

## Slide 1
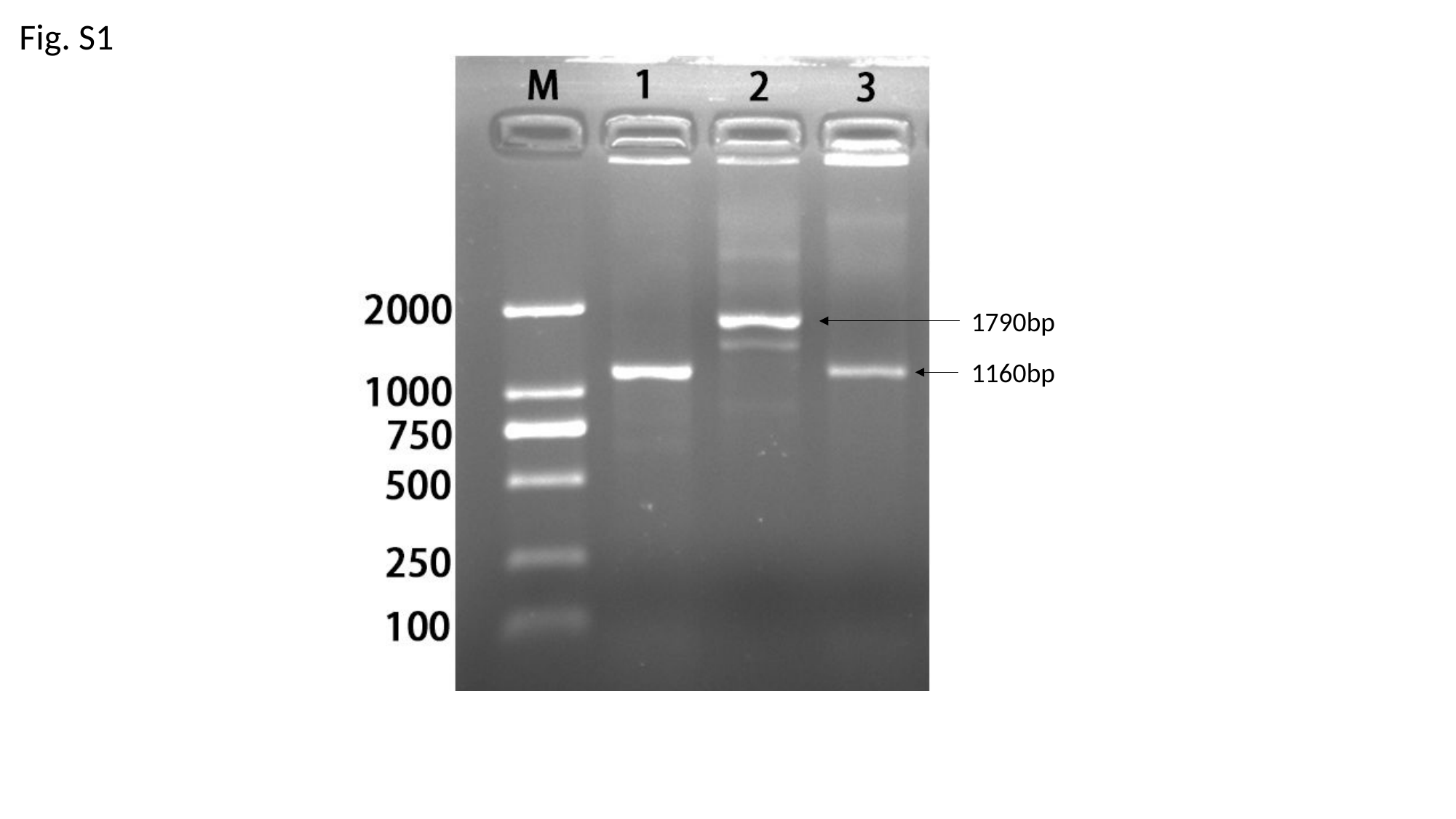

Fig. S1
1790bp
1160bp

## Slide 2
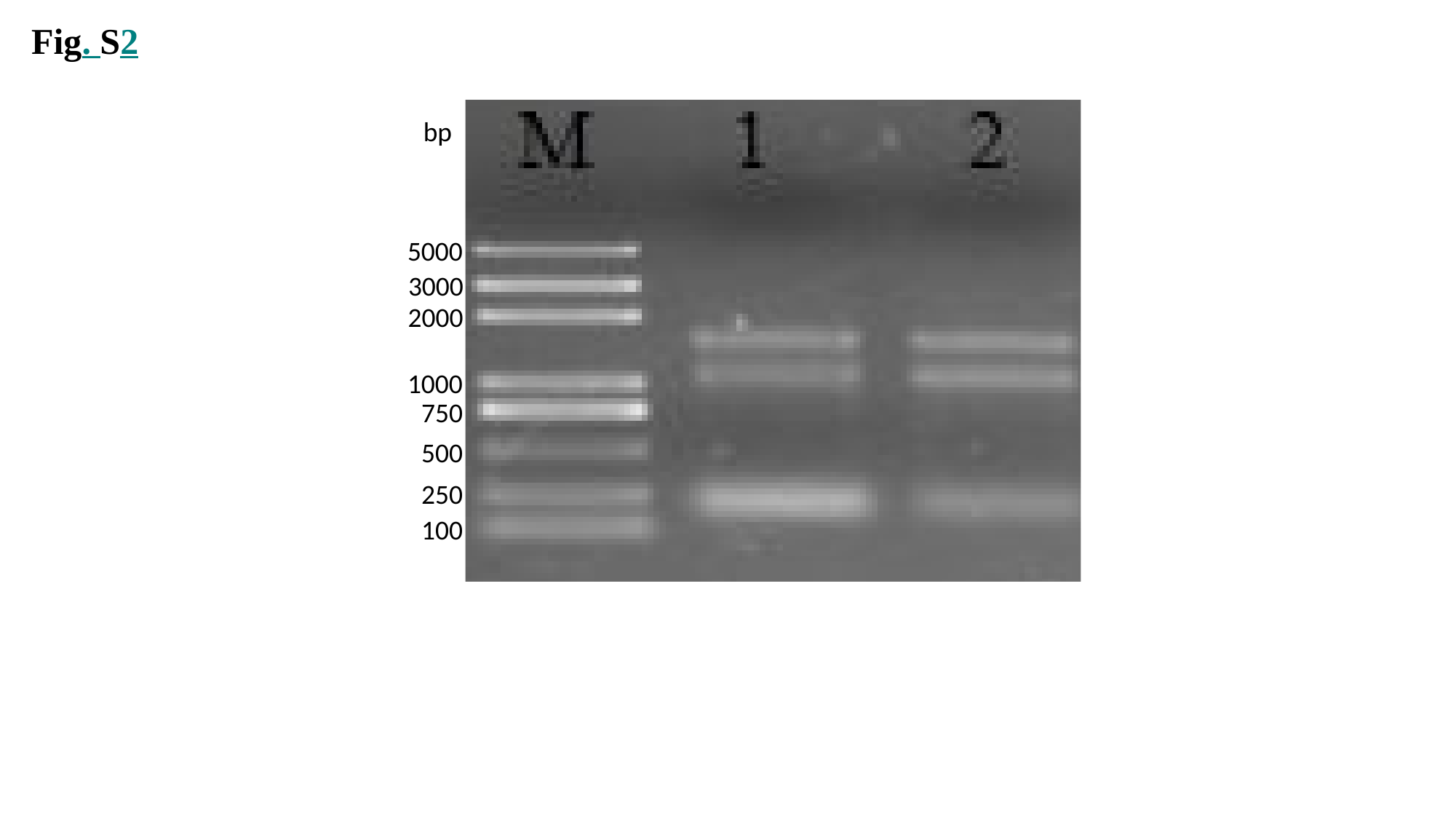

Fig. S2
bp
5000
3000
2000
1000
750
500
250
100

## Slide 3
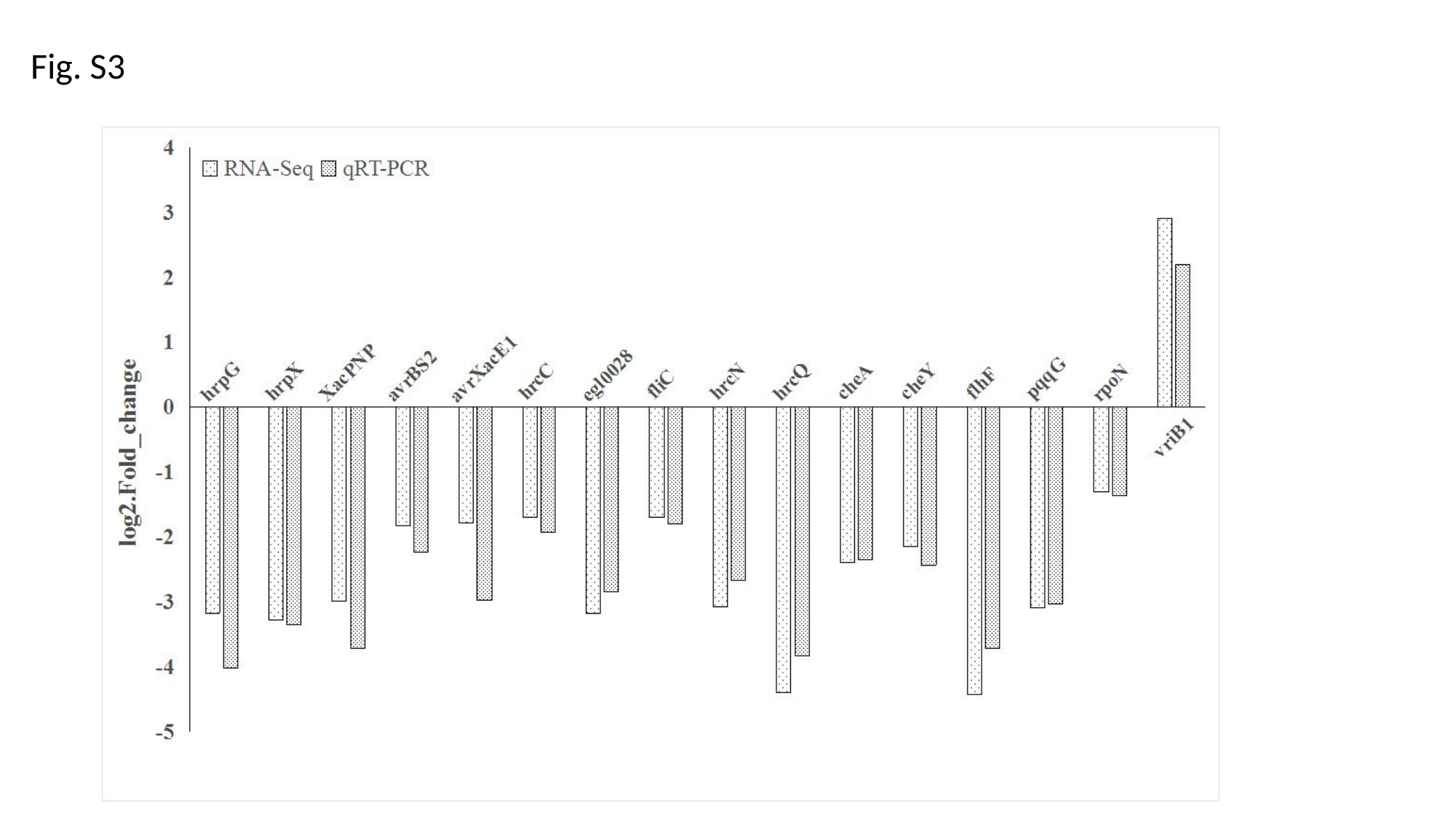

Fig. S3

Supplement: Supplementary file 1 [file genes-10-00340-s001.zip › Fig and Supplementary/Fig.pptx]
